# Data on cost-optimal Nearly Zero Energy Buildings (NZEBs) across Europe

**DOI:** 10.1016/j.dib.2018.02.038

**Published:** 2018-02-16

**Authors:** Delia D'Agostino, Danny Parker

**Affiliations:** a European Commission, Joint Research Centre (JRC), Directorate C - Energy, Transport and Climate, Energy Efficiency and Renewables Unit, Via E. Fermi 2749, I-21027 Ispra (VA), Italy; b Florida Solar Energy Center, University of Central Florida, USA

## Abstract

This data article refers to the research paper A model for the cost-optimal design of Nearly Zero Energy Buildings (NZEBs) in representative climates across Europe [1]. The reported data deal with the design optimization of a residential building prototype located in representative European locations. The study focus on the research of cost-optimal choices and efficiency measures in new buildings depending on the climate. The data linked within this article relate to the modelled building energy consumption, renewable production, potential energy savings, and costs. Data allow to visualize energy consumption before and after the optimization, selected efficiency measures, costs and renewable production. The reduction of electricity and natural gas consumption towards the NZEB target can be visualized together with incremental and cumulative costs in each location. Further data is available about building geometry, costs, CO_2_ emissions, envelope, materials, lighting, appliances and systems.

**Specifications Table**

**Table:** 

Subject area	*Engineering*
More specific subject area	*Building modelling*
Type of data	*Data processed using the software BEopt*
How data was acquired	*Data collected from different sources for the model set up (e.g. IWEC weather data files, Eurostat cost data, market surveys, literature, available information on technological measures, Standards), then processed by BEopt*
Data format	*.BEopt*
Experimental factors	*No pretreatment*
Experimental features	*Data of performance calculations and dynamic simulation modelling of new residential buildings in European locations*
Data source location	*European Member States*
Data accessibility	*Data are provided in*Supplementary materials*directly with this article*

**Value of the data**

• The data provide quantitative information on cost-optimal NZEBs related to new residential buildings across Europe;
• The data give insight on cost-effective technological measures for NZEBs in different European climates;
• The data relate implemented energy efficiency solutions for envelope, appliances, and systems for building design optimization;
• The data give information on modelled energy consumption, renewable production, potential energy savings, and costs in European locations;
• The data can be used for comparison with other NZEBs or further analysis;
• The data support energy efficiency and energy policies implementation at European level [2].

## 1 Data

As foreseen by the recast of the Energy Performance of Buildings (EPBD) Directive [2], all new buildings have to be nearly zero-energy (NZEBs) by the end of 2020 [3]. NZEBs combine efficiency measures and renewable production considering cost-optimal levels of minimum energy performance requirements [4], [5]. The shared data are the building simulations files carried out using the software BEopt. These data identify the optimal building design to reach the NZEBs performance at the lowest cost in different European locations.

Data refer to new residential buildings and include the main building characteristics starting from its general description. In the files, more details are available on efficiency measures, envelope, systems, technologies, lighting, renewables, and costs. The data comprise energy consumption, energy savings and implemented measures in the base and optimized building configurations.

## 2 Experimental design, materials and methods

A simulation-based optimization model has been developed to derive the most cost-effective combination of energy efficiency and renewable energy measures for a residential new building prototype. The methodology and the research assumptions are reported in Section 2 of [1].

BEopt is an optimization software that uses a sequential search technique to optimize the building design starting from a base configuration. It refers to EnergyPlus and TRNSYS to carry out the dynamic simulations of the building. EnergyPlus calculates hourly household heating, cooling, water heating and appliance loads, while TRNSYS estimates the renewable energy production for PV and solar water heating.

**Fig. 1:**
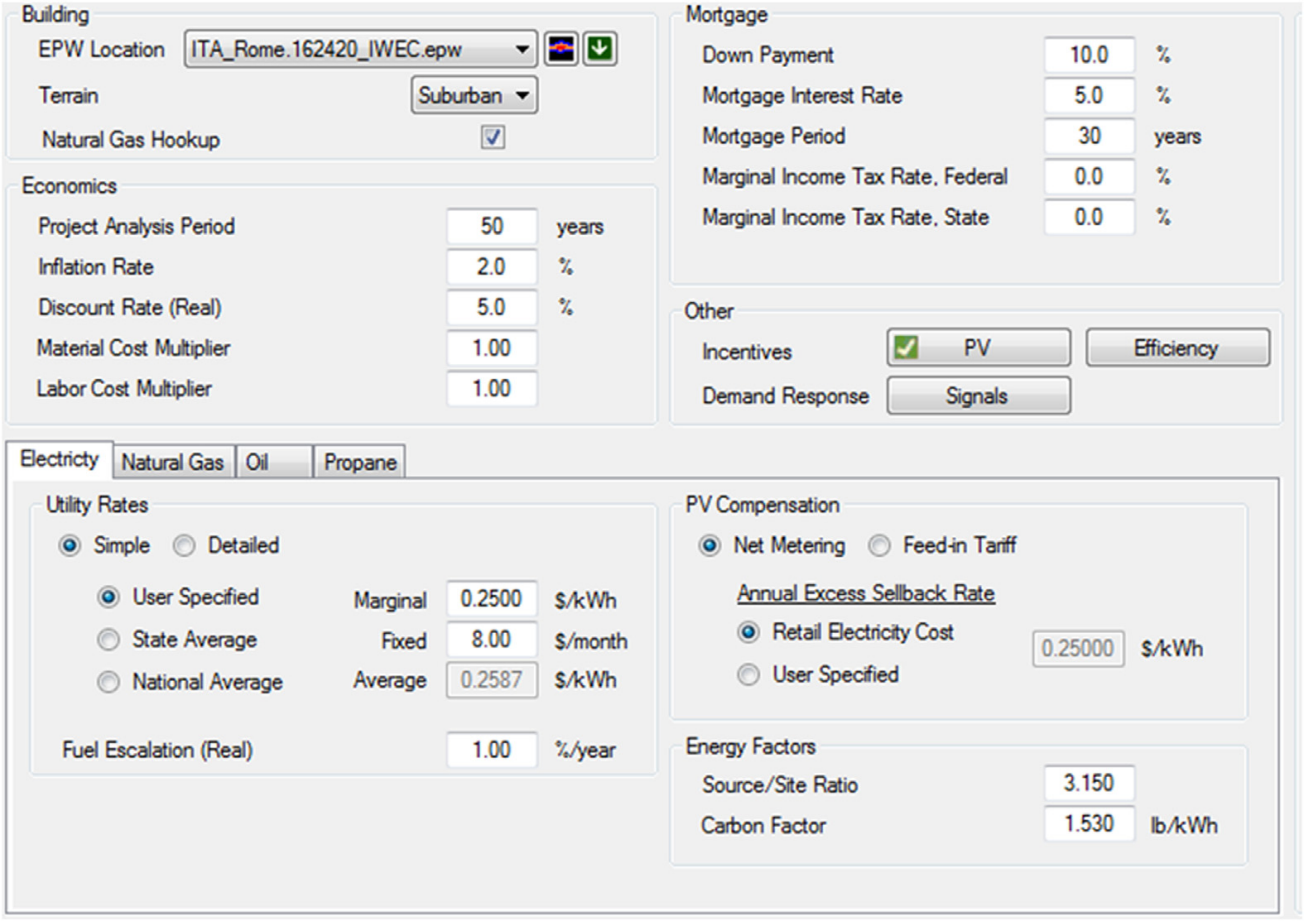
An example of data model input parameters.

The shared data relate to the simulation files of some of locations where the building has been located: Milan, Lisbon, and Stockholm. These locations represent the European climatic variability of a mild, warm, and cold climate. Hourly International Weather for Energy Calculations (IWEC) data files have been included in the model. Modifying the site location from the input parameters (Fig. 1), other cities can be simulated.

The modelled building prototype and its main properties are detailed in Table 5 of [1], while the economic parameters and assumptions are in Section 2.3 of [1].

Data include a library of energy efficient options, related to envelope, appliances and systems. This comprises technical features as well as costs, life expectancy, operation, maintenance, and replacement costs [6], [7], [8]. The potential impact of climate change on the estimated cooling loads has been accounted for in the calculations as explained in Section 2.4 of [1].

**Fig. 2:**
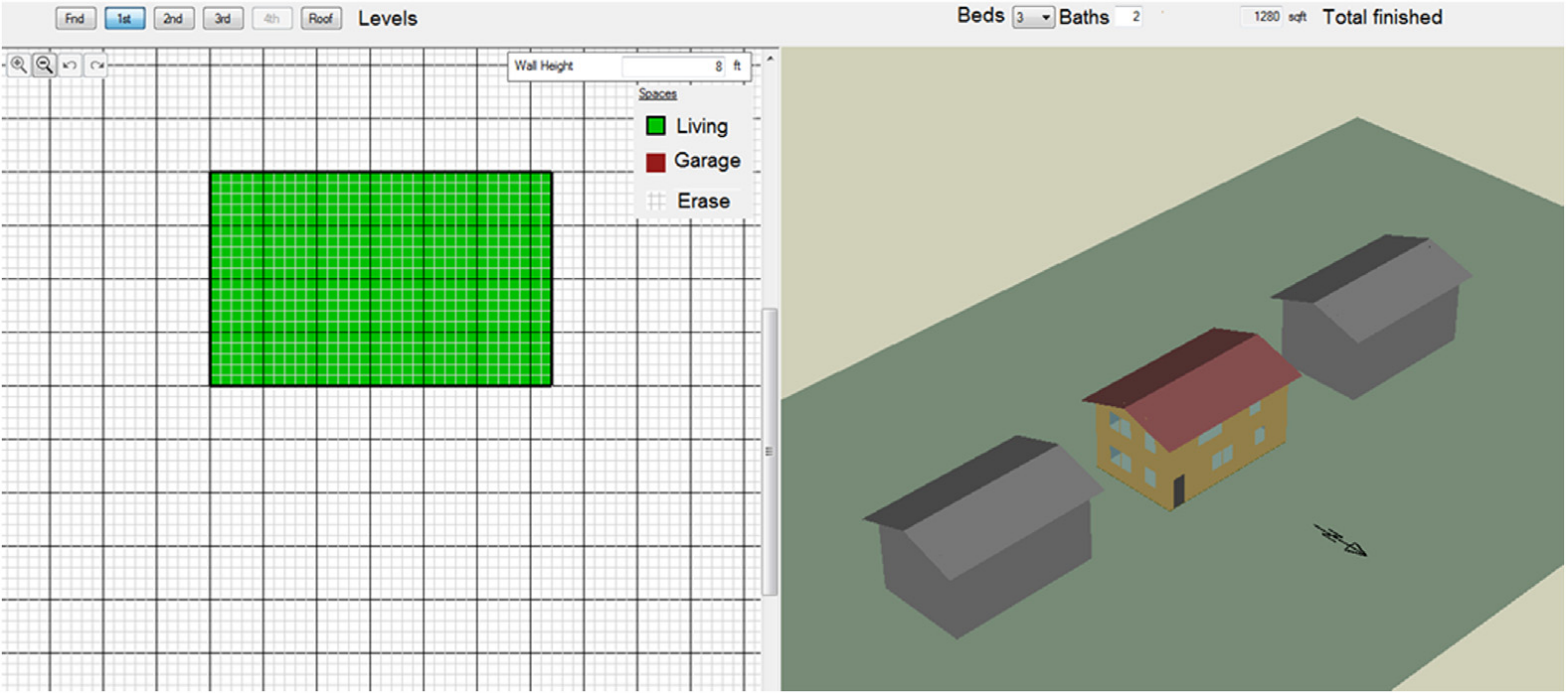
The building geometry.

The shared data include both the model input and output. Main data input relate the building set up, climatic conditions, efficiency options, economic and energy parameters (Fig. 1). Further data is available on building geometry, costs, materials, lighting, appliances, envelope, and systems. Fig. 2 shows how the integration with SketchUp simplifies the creation of the building model.

**Fig. 3:**
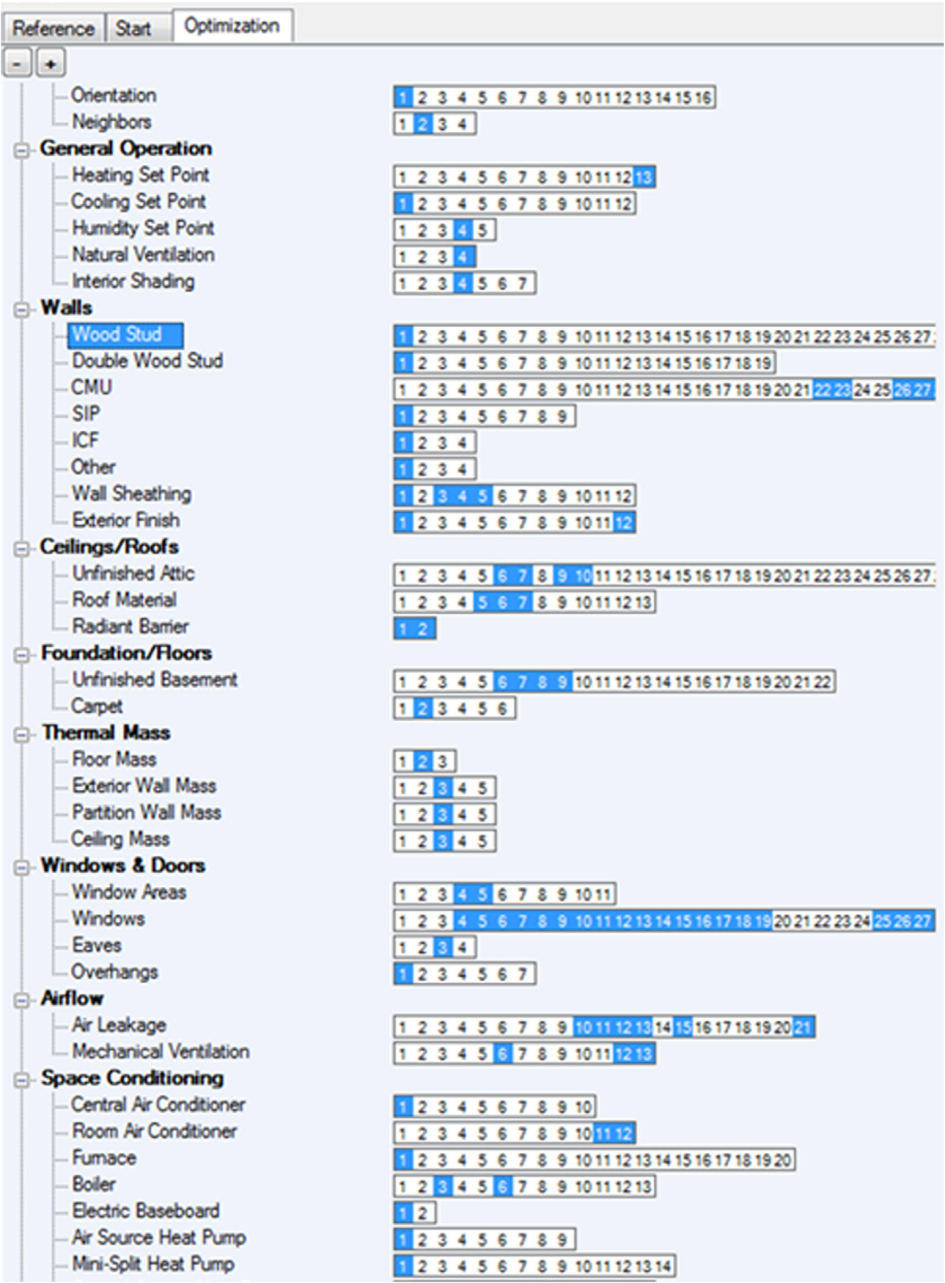
Efficiency options definition.

Fig. 3 shows the available selection of some efficiency options related to: building orientation, walls, ceilings, roofs, foundation, thermal mass, windows, airflow, and space conditioning. For each of them, different technical and economical data have been defined and are available within the provided files.

Data outputs can be visualized in different forms: energy consumption, energy savings, selected efficiency measures, costs and renewable production. For each location, the software ran around 2000 simulations in about 50 iterations to reach the final target.

**Fig. 4:**
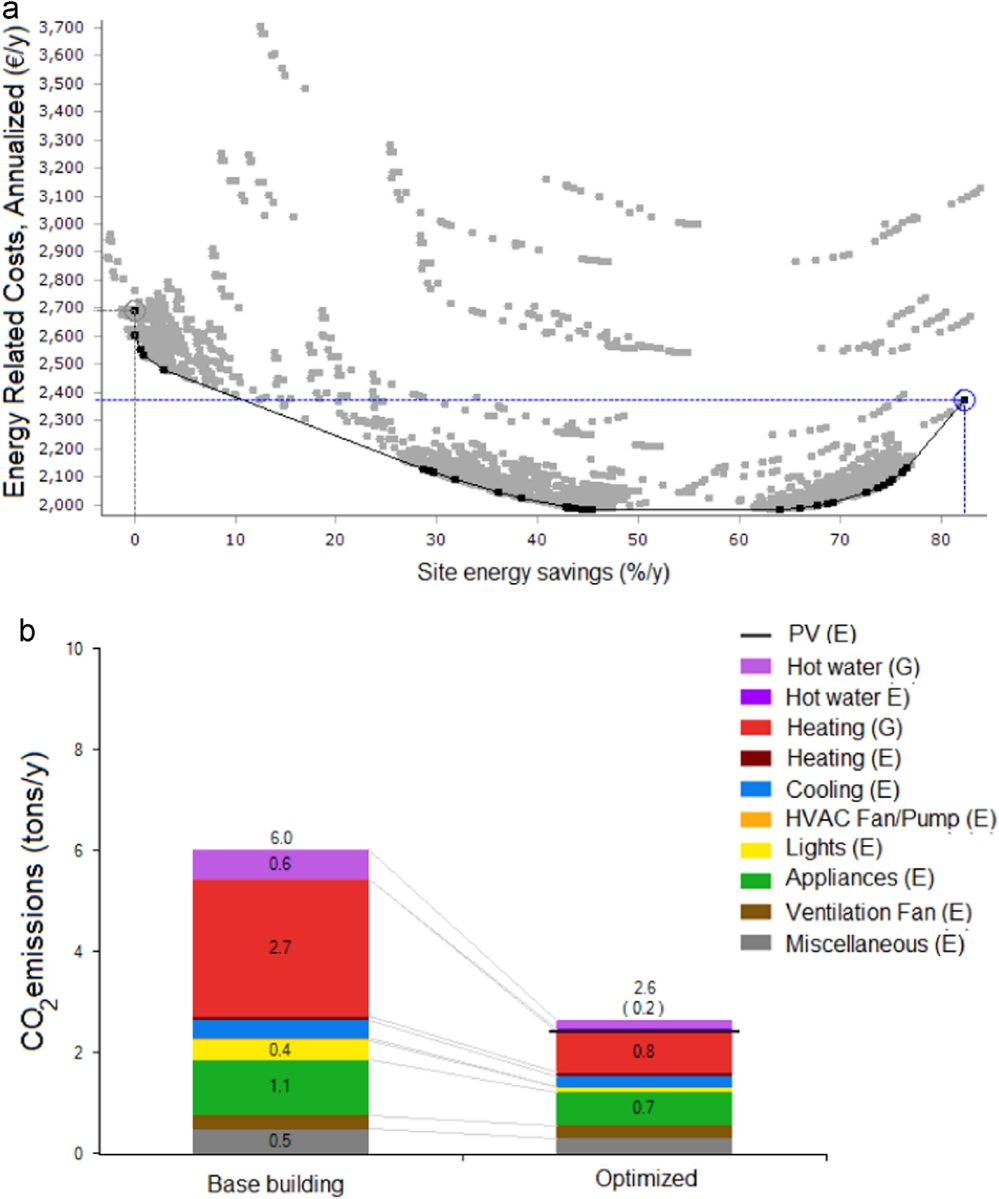
Data processed for the Milan case showing: a) annualized energy related costs vs site energy saving, b) CO_2_ emission reduction between the base and the optimized building for different energy uses, G= gas, E= electricity).

Table 1 reports some parameters that can be arranged in graphs. An example of data visualization is shown in Fig. 4 in relation to the Milan case.

**Table 1:** Possibilities of graph visualization on X and Y axis.

Y-AXIS	X-AXIS
Annualized energy related costs	Site energy consumption
Net present value	Site energy savings
Life-cycle cost	Source energy consumption
Payback time	Source energy savings
Modified internal rate of return	CO_2_ emissions
Source cost of energy	CO_2_ savings

Provided data allow the identification of the NZEB target within the cost-optimal curve that reports global costs (€/m^2^) and energy consumption (kW h/m^2^y). Incremental and cumulative costs in each location can be visualized as well. The reduction of electricity and natural gas consumption towards NZEBs can be derived from the data.

Data comparison can be made before and after the building optimization in relation to the following parameters: electricity, natural gas, oil, propane, CO_2_ emission, heating /cooling loads.
