# Supplementary material for: Data on cost-optimal Nearly Zero Energy Buildings (NZEBs) across Europe
Source: Data Brief. 2018 Feb 16;17:1168–74. doi: 10.1016/j.dib.2018.02.038 (PMC5988456; doi:10.1016/j.dib.2018.02.038)
Supplement: Supplementary file 1 — Transparency document [file mmc1.pdf]

Conflict of Interest and Authorship Conformation Form

Please check the following as appropriate:

- [x] All authors have participated in (a) conception and design, or analysis and interpretation of the data; (b) drafting the article or revising it critically for important intellectual content; and (c) approval of the final version.

- [x] This manuscript has not been submitted to, nor is under review at, another journal or other publishing venue.

- [x] The authors have no affiliation with any organization with a direct or indirect financial interest in the subject matter discussed in the manuscript

- [ ] The following authors have affiliations with organizations with direct or indirect financial interest in the subject matter discussed in the manuscript:

Author's name	Affiliation
Delia D'Agostino	JRC - Joint Research Centre
Danny Parker	Florida Solar Energy Centre
